# Genotoxic effects in island populations of Cartagena de Indias, Colombia due to environmental exposure to mercury and cadmium

**DOI:** 10.12688/f1000research.154617.3

**Published:** 2025-01-17

**Authors:** Eder Cano-Pérez, Doris Gómez-Camargo, Dacia Malambo-García

**Affiliations:** 1 Doctorado en Medicina Tropical, Faculty of Medicine, Universidad de Cartagena, Cartagena, Bolívar, Colombia; 2 Grupo de Investigación UNIMOL, Faculty of Medicine, Universidad de Cartagena, Cartagena, Bolívar, 13001, Colombia

**Keywords:** Cadmium, DNA damage, Genotoxicity, Mercury, Heavy metals

## Abstract

**Background:**

Contamination of Cartagena Bay, Colombia with heavy metals such as mercury (Hg) and cadmium (Cd) presents a major environmental and public health concern, particularly for human communities residing on nearby islands and coastal areas. These populations face enhanced exposure risks owing to their traditional fishing practices and continuous interactions with polluted marine environments. This study aimed to evaluate the genotoxic effects of environmental exposure to Hg and Cd in populations from the island zone of the Cartagena district, Bolívar.

**Methods:**

Ninety-four individuals from the four island communities (study group) and 30 individuals from the urban area of Cartagena (control group) participated in this study. The blood samples were collected to measure total mercury (T-Hg) and Cd concentrations, and a Buccal Micronucleus Cytome (BMCyt) assay was used to evaluate exposure effects.

**Results:**

Cadmiun levels in the blood of the study group were within the normal range and comparable to those of the control group (p > 0.05). However, the study group exhibited significantly higher T-Hg levels (7.34 μg/L) compared to the control group (2.01 μg/L), surpassing the accepted limit. Moreover, the study group showed a higher incidence of DNA damage and cell death biomarkers (p < 0.05). Additionally, significant correlations were observed between total blood Hg levels and the frequencies of micronuclei, karyorrhexis, and karyolysis.

**Conclusion:**

These results suggest that island populations of Cartagena are exposed to high levels of Hg and exhibit genotoxic damage, indicating a problem that must be addressed.

## 1. Introduction

The significance of genotoxic biomonitoring studies lies in their ability to assess and reveal the harmful effects of various physical and chemical agents on population DNA. The most-studied genotoxic agents are pesticides, ^1^ petroleum derivatives, ^2^ drugs, ^3^ and heavy metals. ^4^ In particular, the genotoxic effects of heavy metals such as mercury (Hg) and cadmium (Cd) have been of special interest because of their known mutagenic effects on populations occupationally or environmentally exposed to these metals. ^4^

These metals can cause direct or indirect damage to the structure of genetic material, leading to carcinogenic effects. ^4^^,^^5^ To evaluate these impacts, biomonitoring can be conducted using various human biological samples, including blood, urine, hair, and semen. ^1^^,^^4^^,^^6^ Additionally, their effects can be assessed in different cell lines and derivatives, such as leukocytes, red blood cells, sperm, and buccal mucosa cells. ^1^^,^^4^^,^^7^^,^^8^

Cytogenetic biomarkers have been used in environmental surveillance studies to evaluate human exposure to potentially genotoxic agents. The buccal micronucleus cytome assay (BMCyt) is a suitable and noninvasive method for quantifying DNA damage, chromosomal instability, and cell death in the mucosal tissue. ^9^^,^^10^ Notably, biomarkers measured using this assay have been linked to a heightened predisposition to specific types of cancers. ^10^

The Bay of Cartagena, Colombia, faces severe heavy metal contamination owing to the inflow of sediments from the Magdalena River through the Canal del Dique, which transports approximately 2000 tons of sediments daily from the vast mining and industrial areas within the country. This influx is the primary source of contamination in the Bay. ^11^ Recent ecological studies conducted in this region have highlighted elevated levels of various metals, including Hg and Cd, in sediments of the Bay of Cartagena. ^11^^,^^12^ Moreover, studies have reported elevated Cd levels in oysters ^13^ and significant Hg levels in diverse marine and terrestrial organisms, such as fish, ^14^^,^^15^ crustaceans, ^16^ mollusks, ^17^ and birds, ^18^ within this water body.

Elevated levels of Hg have been detected in the hair and blood of residents of certain island communities in cartagena. ^16^^,^^17^^,^^19^ Owing to their traditional fishing activities and constant interactions with affected marine areas, these populations face heightened risks of exposure to these agents, which enter the human body through the digestive, respiratory, and cutaneous pathways. However, there is a gap in the research regarding the effects of these metals on the genetic material of exposed populations. Therefore, this study assessed the genotoxic effects caused by the presence of Hg and Cd in the blood of coastal and island populations in the district of Cartagena, Colombia using the BMCyt assay.

## 2. Methods

### 2.1 Study area

**Figure 1.**
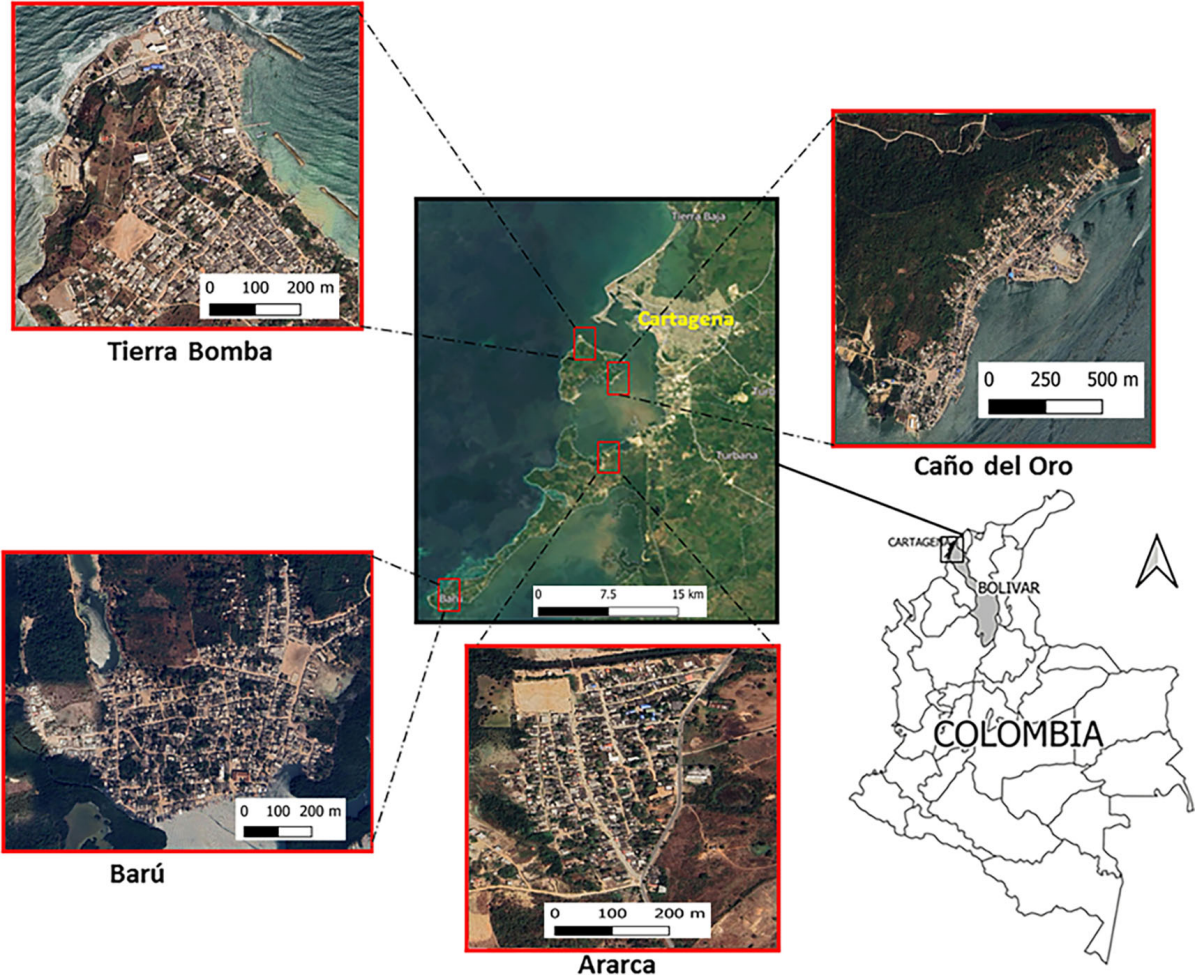
Location of the study sites: Ararca, Barú, Caño del Oro, and Tierra Bomba, Cartagena, Colombia. The satellite images were obtained from Google Earth, Image © 2024 Maxar Technologies.

This was a cross-sectional cohort study conducted between October 2020 and February 2021 in the rural island communities of Ararca, Barú, Caño del Oro, and Tierra Bomba, which are traditional fishing communities located in the district of Cartagena de Indias, Colombia ( Figure 1). Ararca, with a population of 900 inhabitants residing in 219 homes, is situated 15 km away from the urban area of Cartagena and maintains direct interaction with the Bay of Cartagena and Dique Canal; and Barú is a peninsula inhabited by 1922 people living in 448 homes, located 34 km from the urban area of Cartagena. ^20^ Caño del Oro and Tierra Bomba are communities that are part of the island of Tierra Bomba, with populations of 1257 (326 homes) and 1472 inhabitants (298 homes), respectively.

### 2.2 Population and sample

The population analyzed in this study consisted of individuals residing in the communities of Ararca, Barú, Caño del Oro, and Tierra Bomba (5551 inhabitants). To determine the sample size and proportion for each site, the formula for sample estimation was used within a defined population, ^21^^,^^22^ where N represents the total population size (5551 inhabitants); p denotes the expected proportion of individuals with Hg and/or Cd-caused DNA damage, estimated at 50% (0.5); and d2 represents the maximum allowable error of 0.1 (10%).

The total estimated sample size was 94. Subsequently, this number was distributed among the four communities based on the contribution of each community to the total population size. Consequently, the numbers of individuals evaluated in each community were determined to be: 16 from Ararca, 31 from Barú, 22 from Caño del Oro, and 25 from Tierra Bomba, resulting in a combined sample size of 94. Additionally, a control group of 30 individuals residing in the city of Cartagena was included for comparative analysis.

### 2.3 Selection criteria and registration of participants

Participants were registered using a comprehensive survey that collected information on various lifestyle factors, including alcohol and tobacco consumption, dietary habits, water source and treatment, occupational activities, duration of residence, and history of exposure to genotoxic agents, such as chemicals or radiation.

Based on the survey data, individuals included in the study were required to have resided in the study area for more than three years, with no history of alcoholism, smoking, cancer, or chronic degenerative diseases within the six months prior to the survey. Additionally, they should not have undergone any pharmacological treatment and should not have a history of prolonged exposure to any other known chemical or physical genotoxic agent. Failure to meet any of these criteria was considered exclusion criterion.

The inclusion criteria for individuals in the control group were the same as those in the study group except that they should not belong to any of the communities under study. Therefore, individuals residing within the urban area of Cartagena were consulted to ensure a match in sex and age proportions with the study group.

Every participant, without exception, provided their consent by signing an informed consent form. The form outlined the objectives of the study and authorized the use of their biological samples for scientific purposes.

### 2.4 Collecting biological samples

The blood samples and buccal mucosal cells were collected from all participants included in the study. To analyze the levels of Hg and Cd, 3–6 mL of the peripheral blood was drawn into EDTA-containing tubes and stored on ice. For the BMCyt assay, buccal mucosal cell samples were obtained by gently swabbing the inner cheeks with a cotton swab. These cells were then suspended in a cold saline solution (0.9%). Before sample collection, the participants were instructed to rinse their mouths with bottled drinking water provided by the researchers. Subsequently, the samples were transported to the UNIMOL Laboratory at the University of Cartagena for further analysis.

### 2.5 Analysis of heavy metals in the blood

Total mercury (T-Hg) and Cd levels in the blood were determined at the Laboratory of Toxicology and Environmental Management at the University of Córdoba within the first 48 h. Two milliliters of blood were transferred to a 100 mL Teflon vessel, to which 8 mL of concentrated nitric acid and 2 mL of hydrogen peroxide (30%) were subsequently added. The vessels were sealed and digested in an Ethos Touch microwave oven at a temperature of 280 °C, with a pressure of 80 bars and a power of 1400 W for 30 minutes. Once cooled, the sample volumes were adjusted with deionized water to a final volume of 70 mL for T-Hg analysis and 500 μL of the sample adjusted to a final volume of 1 mL for Cd analysis. T-Hg analyses in the blood were performed using Cold Vapor Atomic Absorption Spectrophotometry (CVAAS; Thermo Scientific ^TM^, model iCE, series 3000) at a wavelength of 253.7 nm and detection limit of 0.40 μg/L. ^23^ Blood Cd analyses were conducted using Graphite Furnace Atomic Absorption Spectrometry (GFAAS, Thermo Scientific iCE 3000 Series equipped with a graphite furnace) with a detection limit of 0.20 μg/L. ^24^ Notably, Cd analysis was not possible for four individuals because of sample scarcity. Concentration units for both metals were determined in micrograms per liter. The exposure limits for T-Hg and Cd in the blood were established following the guidelines of the World Health Organization (WHO) ^25^ and the Centers for Disease Control and Prevention (CDC), ^26^ respectively; both of which specify an allowable limit of up to 5 μg/L in the blood for these metals.

### 2.6 Buccal micronucleus cytome assay

After buccal massage, the cells were suspended in 15-mL centrifuge tubes containing 5 of 0.9% saline solution (w/v). Upon arrival at the laboratory, samples were centrifuged at 1500 RPM for 10 min. The settled buccal cells were washed twice with saline solution and once with Carnoy’s fixative (methanol and glacial acetic acid, 3:1) under the same centrifugation conditions. Subsequently, 80–100 μL of cell suspension were carefully applied to angled slides to ensure optimal cell spreading. ^2^ The fixed cells were stained using the Feulgen method, following the recommendations of Thomas et al. ^10^ In summary, the slides were first treated in a 50% and 20% ethanol solution for 1 minute at room temperature, followed by immersion in distilled water for 2 minutes. Next, the slides were exposed to 5 M HCl for 30 minutes. A slide not treated with HCl served as a negative control during this step. After this treatment, the slides were rinsed with running water for 3 minutes and then immersed in Schiff’s reagent (Sigma-Aldrich, St. Louis, MO, USA) for 1 hour in complete darkness at room temperature. Following this, the slides were rinsed for 5 minutes with running water and then with distilled water. Finally, the slides were immersed in 0.2% (w/v) Light Green solution (Sigma-Aldrich, St. Louis, MO, USA) for 20 to 30 seconds, rinsed with distilled water, and allowed to air dry for 15 minutes at room temperature.

**Figure 2.**
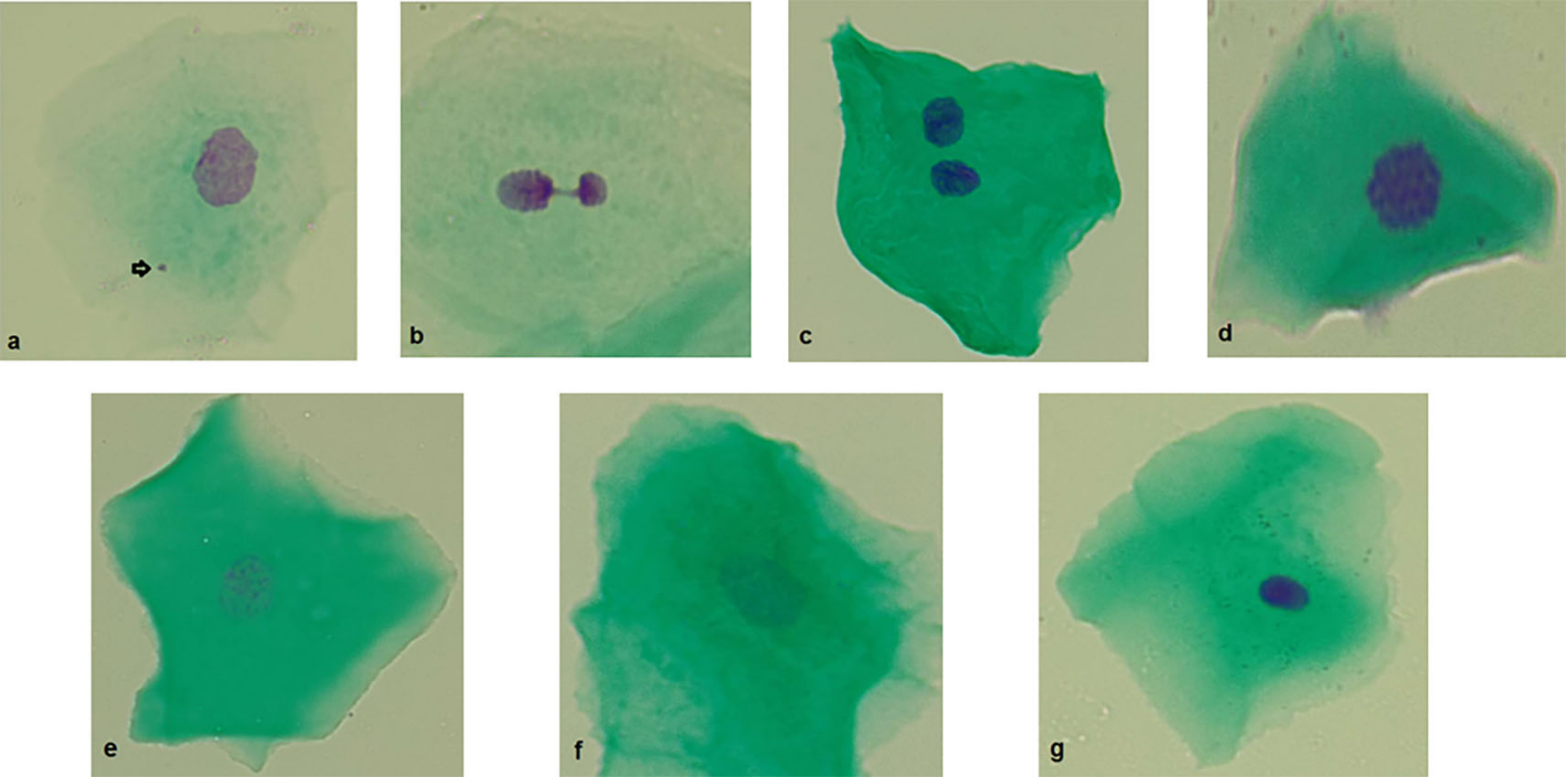
Photographs (100×) of different anomalies in exfoliated cells from the buccal mucosa. a) Micronucleus. b) Nuclear bud. c) Binucleated cell. d) Condensed chromatin. e) Karyorrhexis. f ) Karyolysis. g) Pyknosis. Original Images from this study.

Stained slides were examined using an optical microscope (Leica Microsystems, Germany), with 1000 cells/individual visualized and analyzed at 100× magnification. Nuclear abnormalities such as DNA damage (micronuclei and nuclear buds), cell death indicators (condensed chromatin, pyknosis, karyorrhexis, and karyolysis), and cytokinesis defects (binucleated cells) were assessed following the recommendations of Thomas et al. ^10^:

Micronucleated cells: micronucleated cells possess a main nucleus as well as one or more micronuclei. Micronuclei are generally round or oval and exhibit a staining intensity comparable to that of the main nucleus. They typically measure between 1/3 and 1/16 the diameter of the main nucleus and are situated within the cellular cytoplasm ( Figure 2a).

Nuclear buds: the main nucleus exhibits a sharp constriction that forms a bud, which remains attached to it. This bud has a staining intensity similar to that of the main nucleus and its diameter ranges from one-quarter to one-half of the nuclear diameter ( Figure 2b).

Binuclead cell: Cells contain two main nuclei of similar size and staining intensity. The nuclei are usually very close and may touch each other and usually have the same morphology as that observed in normal cells ( Figure 2c).

Condensed chromatin: the nucleus shows areas of aggregated chromatin, with distinct regions that are more intensely stained. Additionally, the nucleus exhibits a striated pattern ( Figure 2d).

Karyorrhexis: The nucleus exhibits extensive aggregation of chromatin, which can be observed in substantial clusters. Additionally, nuclear fragmentation may be evident ( Figure 2e).

Karyolysis: the nucleus is depleted of DNA, as indicated by its lack of staining with the Feulgen method (the nucleous apparent as a ghost-like image) ( Figure 2f).

Pyknosis: the cell has a small, shrunken nucleus that is uniformly and intensely stained. The diameter of this nucleus is approximately 1/3 to 2/3 of the diameter of a normal nucleus ( Figure 2g).

### 2.7 Data analysis

The normality of the variables was assessed using the Kolmogorov–Smirnov test or Shapiro–Wilk test to determine whether parametric or non-parametric analysis was appropriate. The Shapiro-Wilk test is more appropriate for small sample sizes (N ≤ 50), while the Kolmogorov-Smirnov test is used for larger sample sizes (N > 50). Both the Shapiro-Wilk and the Kolmogorov-Smirnov tests compare the scores in the study sample with a normally distributed set of scores with the same mean and standard deviation; their null hypothesis is that sample distribution is normal. Therefore, if the test is significant (p < 0.05), the sample data distribution is non-normal. ^28^

In our study, the variables did not follow a normal distribution (p < 0.05). Therefore, the results are reported as medians and interquartile ranges (IQR: 25 ^th^–75 ^th^ percentile). Significant differences between groups were examined using the Mann–Whitney U test or Kruskal–Wallis test for multiple comparisons, followed by post-hoc Dunn’s test for each pairing. The Mann-Whitney U test and the Kruskal-Wallis test are non-parametric tests that assume a non-normal distribution of data. The Mann-Whitney U test can only deal with the comparison between two samples, whereas the Kruskal-Wallis test can handle multiple samples. Furthermore, post-hoc tests such as Dunn’s test enable us to identify which specific groups exhibit statistical differences. ^29^

Finally, correlations among blood metal levels, quantitative demographic variables, and BMCyt parameters were assessed using Spearman’s coefficient (a non-parametric test for establishing correlations). All analyses were performed using the statistical software SPSS v.19.0 (IBM Corp, Armonk, NY, USA). Statistical significance was defined as p < 0.05.

## 3. Results

The main demographic characteristics of the study population are summarized in Table 1. The median age of the study group was 31.5 years (IQR: 23–42.25), comprising 69 females (73.4%) and 25 males (26.6%). No significant differences in the median age were observed between the study and control groups (p = 0.178). Regarding education, four individuals in the study group were illiterate, 33% had not completed primary or secondary education, and only two held university degrees. In terms of occupation, 56.38% were homemakers, 30.85% were employed, 9.57% were students, and 3.19% were unemployed. Among those employed, 34.48% worked in tourism, 13.79% in commerce, and 6.89% were fishermen. Other occupations constituted the remaining 44.84%. Approximately 78.72% obtained water from rain or ponds because of the lack of access to a piped water supply system. In terms of diet, fish was the primary protein source, with 40.42% consuming it daily and 45.75% consuming it at least once per week. All individuals followed to a balanced diet consisting of rice, flour, legumes, fruits, and vegetables.

**Table 1.** Demographic characterization of the study population.

Demographic variables	Control group (n=30)	Study group				
		Ararca	Barú	Caño del oro	Tierra Bomba	Total
		n=16	n=31	n= 22	n=25	n= 94
Age (years)	**28 (21.0–41.0)**	35 (24–45)	32 (22–42.7)	32 (22–48)	31.5 (23–41.5)	** 31.5 (23–42.2)**
Duration of residence (years)	**24.5 (8.7-43.0)**	26 (15.0 – 40.0)	27 (14.0 – 40.0)	28 (14.7 – 40.2)	29 (15.0 – 40.0)	**27 (15.7 – 40.0)**
Sex						
Female	**22 (73.3%)**	13	16	18	22	**69 (73.4%)**
Male	**8 (26.7%)**	3	15	4	3	**25 (26.6%)**
Educational level						
None	**0**	1	2	1	0	**4 (4.2%)**
Elementary or incomplete secondary education	**0**	6	11	11	3	**31 (33%)**
High school graduate	**12 (40.0%)**	7	14	8	16	**45 (47.9%)**
University studies	**17 (60.0%)**	2	4	2	6	**14 (14.9%)**
Occupation						
Homemaker	**1 (4.0%)**	12	10	14	17	**53 (56.38%)**
Employed	**17 (56.0%)**	4	17	2	6	**29 (30.85%)**
Unemployed	**0**	0	0	2	1	**3 (3.19%)**
Student	**12 (40.0%)**	0	4	4	1	**9 (9.57%)**
Alcohol consumption						
Consumer *	**2 (7.0%)**	1	7	1	0	**9 (9.57%)**
Non-consumer	**28 (93.0%)**	15	24	21	25	**85 (90.43%)**
Fish consumption						
Non-consumer	**1 (4.0%)**	1	0	2	0	**3 (3.19%)**
Occasional (< once per week)	**17 (56.0%)**	5	3	1	1	**10 (10.64%)**
Weekly	**12 (40.0%)**	7	13	10	13	**43 (45.75%)**
Daily	**0**	3	15	9	11	**38 (40.42%)**
Source of drinking water						
Tanker or rainwater	**0**	0	29	21	24	**74 (78.72%)**
Aqueduct or commercial	**30 (100%)**	16	2	1	1	**20 (21.28%)**

* Non-alcoholic consumers with a low level of consumption according to the National Institute of Alcohol Abuse and Alcoholism - NIAAA.

### 3.1 Analysis of metals heavy in blood

Blood concentrations of T-Hg and Cd are presented in Table 2. The median T-Hg level was significantly higher in the study group than in the control group (p < 0.001). Furthermore, the averages exceeded the permissible limits set by the WHO for populations environmentally exposed to Hg, both within communities and overall. When comparing different communities, Barú exhibited significantly higher levels as compared to Ararca and Caño del Oro.

**Table 2.** Mercury and cadmium concentrations in the blood of the study population.

Heavy metals	Control group (n=30)	Study group					Permissible limits (μg/L)
		Ararca (n=16)	Barú (n=31)	Caño del Oro (n=22)	Tierra Bomba (n=25)	Total (n=94)	
T-Hg (μg/L)	1.61 (1.18–2.69) **b**	6.47 (2.45–8.30) **a**	10.03 (6.94–13.19) **c**	5.50 (2.56–8.85) **a**	7.26 (5.55–13.51) **ac**	7.39 (4.54–11.20) **a**	5.0
Cd (μg/L)	0.49 (0.33–0.85) **b**	-	0.60 (0.28 – 1.50)	0.60 (0.34 – 1.28)	0.40 (0.33 – 1.67)	0.43 * (0.33–1.02) **b**	5.0

Different letters indicate statistically significant differences (p < 0.05). The median Cd concentration in the study group was calculated on a basis of 20 individuals (Ararca = 1 [1.06 μg/L], Barú = 5, Caño del Oro = 6, Tierra Bomba = 8).

* Median calculated based on a total of 20 individuals (Ararca = 1, Barú = 5, Caño del Oro = 6, Tierra Bomba = 8).

Analysis of blood Cd levels revealed no significant differences between the study and control groups. Notably, 77.7% (70/90) of the examined individuals exhibited values below the detection limit for Cd in the blood, according to the employed methodology (0.2 μg/L). Among the 20 individuals in whom Cd was detectable, the levels ranged from 0.2 to 2.99 μg/L (median = 0.43 μg/L). This suggests that none of the participants exceeded the Cd limit set by the CDC (5 μg/L). Additionally, an inverse relationship was observed between blood Cd and T-Hg concentrations in this group, although it was not statistically significant (Rho = -0.438; p = 0.053).

Comparisons of demographic variables potentially influencing the blood levels of T-Hg and Cd within the study group, such as sex, age, duration of residence in the area, fish consumption, and source of drinking water, are presented in the Supplementary Material ( **Tables S1 and S2**). The analysis revealed that men and individuals consuming fish daily exhibited significantly higher blood T-Hg levels (p < 0.05) ( **Table S1**). Spearman’s correlation analyses indicated significant associations between blood T-Hg levels and age (Rho = 0.260; p = 0.012), duration of residence in the area (Rho = 0.374; p < 0.001), and fish consumption (Rho = 0.415; p < 0.001). No differences between the groups or correlations between different variables and blood Cd levels were observed (p > 0.05) ( **Table S2**).

### 3.2 BMCyt parameters

Table 3 presents a comparison of the BMCyt biomarkers between the control and study groups. Significantly higher frequencies of all BMCyt assay biomarkers (except pyknosis) were observed in the study group than in the control group (p < 0.05). Site-specific comparisons revealed that the frequency of nuclear alterations indicative of cell death (condensed chromatin, karyorrhexis, and karyolysis) was generally lower in the Ararca community than in the other communities. Figure 2 outlines each of the reported biomarkers.

**Table 3.** Comparison of BMCyt assay biomarker results.

BMCyt	Control group (n= 30)	Study group				Total (n=94)
		Ararca (n=16)	Barú (n=31)	Caño del Oro (n=22)	Tierra Bomba (n=25)	
Micronucleus	**0.6 (0–1.0)b**	5.0 (3.25–5.75) **a**	6.0 (4.0–7.0) **a**	5.0 (3.0–7.25) **a**	5.0 (4.0–8.0) **a**	** 5.0 (4.0–7.0) *a***
Nuclear Bud	**2 (1.0–3.0)b**	6.0 (4.25–7.0) **a**	5.0 (4.0–7.0) **a**	7.0 (5.0–11.0) **a**	4.0 (3.5–8.0) **a**	**6.0 (4.0–8.0)a**
Binucleated cell	**3.5 (1.75–3.0)b**	7.0 (5.0–10.75) **a**	7.0 (6.0–8.0) **a**	9.0 (5.75–11.25) **a**	8.0 (6.0–10.0) **a**	**8.0 (6.0–9.0)a**
Condensed chromatin	**34.3 (29.0–41.2)b**	42.0 (25.25–45.00) **b**	52.0 (35.0–57.25) **c**	53.0 (40.5–77.5) **c**	44.0 (28.5–63.5) **bc**	**45.0 (35.75–65.0)a**
Karyorrhexis	**16.5 (8.75–41.25)b**	32.5 (17.25–48.00) **b**	74.0 (60.0–88.0) **c**	68.0 (50.0–83.75) **c**	76.0 (41.5–92.5) **c**	**63.5 (41.75–86.0)a**
Karyolysis	**19.0 (8.5–33.25)b**	29.0 (17.25–48.00) **b**	52.0 (17.25–48.00) **c**	36.5 (17.25–48.00) **c**	45.0 (17.25–48.00) **c**	**45.0 (28.0–65.0)a**
Pyknosis	**4.0 (3.7–5.01)a**	3.5 (0.0–7.5) **a**	4.0 (2.0–5.0) **a**	2.0 (0.75–5.25) **a**	5.0 (1.00–6.0) **a**	**4.0 (1.0–6.0)a**

Different letters indicate statistically significant differences (p < 0.05).

Table 4 shows the Spearman’s correlations between blood metal levels and BMCyt biomarkers. Significant positive correlations were observed between T-Hg levels and the frequencies of micronuclei, karyorrhexis, and karyolysis. Interestingly, significant negative correlations were found between Cd levels and the frequency of micronuclei and nuclear buds. Considering that sex and age might significantly influence the parameters measured by the BMN-cyt assay, we evaluated these factors as potential predictors of the frequency of these parameters. Our analysis found no differences between sexes and no significant correlation between age and the parameters tested by the BMCyt assay ( **Table S3 and S4**).

**Table 4.** Spearman’s correlation between blood metal levels and BMCyt assay biomarkers in the study group.

BMCyt	T-Hg in blood (n = 94)		Cd in blood (n = 20)	
	Rho (IC 95%)	p	Rho (IC 95%)	p
Micronucleus	0.377 (0.19 – 0.54)	**< 0.001**	-0.465 (-0.75 – -0.03)	**0.039**
Nuclear Bud	0.195 (-0.01 – 0.38)	0.059	-0.522 (-0.78 – -0.10)	**0.018**
Binucleated cell	0.059 (-0.15 – 0.20)	0.574	-0.280 (-0.64 – 0.19)	0.232
Condensed chromatin	0.139 (-0.07 – 0. 33)	0.181	0.280 (-0.19 – 0.64)	0.231
Karyorrhexis	0.375 (0.19 – 0.54)	**< 0.001**	-0.360 (-0.69 – 0.10)	0.119
Karyolysis	0.393 (0.21 – 0.55)	**< 0.001**	0.119 (-0.53 – 0.34)	0.257
Pyknosis	0.148 (-0.06 – 0.34)	0.155	-0.016 (-0.46 – 0.43)	0.947

## 4. Discussion

Assessment of the genotoxic effects associated with heavy metal concentrations in the blood plays a fundamental role in the primary monitoring of genetic material integrity and the well-being of populations. This study provides the first evidence related to the evaluation of genotoxic effects exerted by the environmental exposure to Hg and Cd using BMCyt assay in the communities of Ararca, Barú, Caño del Oro, and Tierra Bomba, rural island communities in the district of Cartagena de Indias, Colombia.

Our findings revealed that the blood levels of Cd in the study group were within the normal range and comparable to those of the control group. In contrast, residents of these island communities exhibited significantly higher blood concentrations of T-Hg than the control group, surpassing the internationally accepted reference levels set by WHO (5 μg/L). ^25^ Given the well-known mercury contamination issue in fish from the Bay of Cartagena, ^14^^,^^15^ it is plausible that the high consumption of fish is the primary source of these metals in these communities. Accordingly, our analyses indicated that individuals who consumed fish daily had elevated blood Hg levels compared to those who consumed fish less frequently. A significant correlation between fish consumption and Hg concentrations was also identified. These findings are consistent with previous research that reported similar correlations between fish consumption frequency and Hg concentrations in hair and blood among populations in the Colombian Caribbean Coast and other regions of Colombia exposed to this metal. ^16^^,^^19^^,^^30^^–^^34^

Mercury can induce genotoxicity through three main mechanisms. First, it generates free radicals and oxidative stress, which can damage DNA and contribute to cell death events. Second, Hg can interfere with DNA repair by inhibiting the enzyme DNA polymerase, resulting in the accumulation of mutations and the occurrence of carcinogenic events. Finally, Hg inhibits microtubule formation, disrupting normal chromosomal separation and leading to the formation of micronuclei. ^35^^,^^36^

The BMCyt assay revealed significantly higher frequencies of micronuclei and nuclear buds (indicators of DNA damage and genetic instability) in the buccal cells of the study group than in those of the control group (p < 0.05). Additionally, a significant positive correlation was identified between the Hg concentration and micronucleus frequency. Similar findings have been reported for other Colombian populations environmentally exposed to Hg. ^37^ Furthermore, studies utilizing the cytokinesis-block micronucleus assay (CBMNcyt) have reported correlations between micronucleus frequency in lymphocytes and the Hg concentration in the blood of environmentally exposed populations. ^38^^–^^40^ In the current study, the CBMNcyt assay was not used; however, a close positive correlation between micronucleus frequency in BMCyt and CBMNcyt has been demonstrated. ^41^^,^^42^ Therefore, despite methodological differences, our findings are generally similar and consistent with those of multiple reports in the literature, suggesting that Hg is a heavy metal associated with increased DNA damage in exposed populations.

The presence of nuclear buds indicates genetic amplification, implying an increase in the number of copies of specific genome sections. ^43^^,^^44^ This amplification results in more intense expression of genes located in the amplified region. A significant biological implication of the increased frequency of nuclear buds concerning the health of the studied populations is the potential role of gene amplification in cancer development. ^45^

In addition to the formation of micronuclei and nuclear buds, various biomarkers of cell death, including cells with condensed chromatin, karyorrhexis, karyolysis, pyknosis, and other nuclear anomalies, such as binucleated cells (indicative of cytokinesis defects), were assessed. Our findings revealed a significant increase in the frequencies of condensed chromatin, karyorrhexis, and karyolysis in the study group compared to those in the control group. Moreover, notable correlations were identified between T-Hg levels and the frequencies of karyorrhexis and karyolysis. These results align with those of Cruz-Esquivel et al., ^37^ who reported a significant increase in all biomarkers related to cell death and cytokinesis defects in BMCyt among Hg-exposed human populations, in contrast to the non-exposed group. The study also highlighted a significant positive correlation between Hg concentration and karyolysis.

Nuclear alterations indicative of cell death represent apoptotic or necrotic events. Some authors have noted that condensed chromatin and karyorrhexis accompany the early stages of apoptosis (which can lead to subsequent necrotic changes), whereas karyolysis is evident only in the necrotizing cells. ^46^^,^^47^ Nuclear irregularities in oral tissue usually occur in the superficial cell layer as part of the natural process of cell renewal. However, excessive cell death events, particularly apoptosis, beyond normal levels can serve as an indicator of exposure to genotoxic agents. ^46^

Controversially, significant negative correlations were observed between blood Cd levels and the frequency of micronuclei and nuclear buds. It is possible that these results are due to the inverse relationship (marginally significant) found between T-Hg and Cd in blood within this group. Consequently, higher T-Hg concentrations are associated with increased biomarkers of damage in individuals with lower cadmium levels. Moreover, the correlation results may be influenced by the small sample size (n = 20), as the wide confidence intervals, approaching zero, suggest that the data do not provide a precise estimate of the true correlation. This implies that the effect may become non-significant in a larger sample.

In buccal cells, alcohol intake may also be a risk factor for increased frequency of oral anomalies and the development of cancers in the oral cavity, pharynx, and larynx. ^48^ However, our study found no influence of alcohol consumption on any of the BMCyt parameters evaluated. This finding was expected, given that the majority of both exposed and unexposed individuals reported not consuming alcohol, with only 10% being light consumers. Additionally, neither age nor sex influenced the frequency of micronuclei or cell death biomarkers in the studied communities. Similarly, various authors have noted that most human biomonitoring studies conducted using the BMCyt assay have not found a statistically significant influence of age or lifestyle on micronucleus frequency in the study populations. ^42^^,^^49^

### 4.1 Limitations of the study

Limitations of this study include several factors that may have influenced the interpretation of results. Firstly, the sample size, although calculated based on statistical power, may not fully represent the diversity within the island communities of Cartagena. Secondly, while efforts were made to control for confounding variables such as age, sex, and some habits, other environmental and genetic factors (not analyzed in this study) could have influenced the observed genotoxic effects. Additionally, the BMCyt assay, while effective for assessing buccal cell damage, provides a snapshot of genetic damage that may not capture long-term effects. Furthermore, the study’s cross-sectional design limits the ability to establish causal relationships between heavy metal exposure and genotoxic outcomes. Longitudinal studies would provide more robust evidence of the cumulative effects of chronic exposure to mercury on genetic integrity in these vulnerable populations.

## 5. Conclusion

In summary, this study provides the first evidence related to the assessment of the genotoxic effects caused by environmental exposure to Hg and Cd using the BMCyt assay in the island communities of Cartagena de Indias, Colombia. The main findings revealed significantly higher levels of T-Hg in the studied communities than in the control population, which exceeded the established limits. Results from the BMCyt assay indicated a significantly higher frequencies of almost all biomarkers analyzed in the study population than in the control group. Furthermore, correlation analyses indicated an increase in the frequency of micronuclei, karyorrhexis, and karyolysis as blood Hg concentrations increased. These findings suggest that Hg is a potential genotoxic agent in the island populations of Cartagena de Indias, Colombia.

## Ethics and consent

This study was conducted as part of the Basin Sea Interactions with Communities (BASIC-Cartagena) project, a multicenter applied research initiative on the interactions between basins, the sea, and communities in the coastal area of Cartagena. ^27^ Participants from these communities were provided with their individual blood heavy metal results accompanied by pertinent recommendations explained by a healthcare professional. This study was conducted in accordance with the Declaration of Helsinki and was approved by the ethics committee of the Faculty of Medicine at the University of Cartagena in May of 2021 (Approval number 045-2021), adhering to the ethical requirements outlined in Resolution 08430 of 1993 by the Ministry of Health of Colombia.

Every participant, without exception, provided their consent by signing an informed consent form.

## Data Availability

Figshare: Genotoxic effects in island populations of Cartagena de Indias, Colombia due to environmental exposure to mercury and cadmium Supplementary material and dataset, https://doi.org/10.6084/m9.figshare.26373451.v2. ^50^ This project contains the following underlying data:
• Supplementary material (Tables S1, S2, S3 and S4) • Dataset (original raw data) • Participant registration form Data are available under the terms of the Creative Commons Attribution 4.0 International license (CC-BY 4.0).
