# Supplementary material for: Genotoxic effects in island populations of Cartagena de Indias, Colombia due to environmental exposure to mercury and cadmium
Source: F1000Res. 2025 Jan 17;13:946. Originally published 2024 Aug 21. [Version 3] doi: 10.12688/f1000research.154617.3 (PMC11747300; doi:10.12688/f1000research.154617.3)
Supplement: Peer review history

# Reviewer report: Reviewer response for version 3

**DOI:** 10.5256/f1000research.176785.r360145

**Published:** 2025-01-20

**Authors:** Tommaso Filippini

**Affiliations:** 1 University of Modena and Reggio Emilia, Modena, Italy

Authors addressed all comments and the revised manuscript has improved. No further suggestions

Is the work clearly and accurately presented and does it cite the current literature?

Yes

If applicable, is the statistical analysis and its interpretation appropriate?

Yes

Are all the source data underlying the results available to ensure full reproducibility?

Partly

Is the study design appropriate and is the work technically sound?

Yes

Are the conclusions drawn adequately supported by the results?

Yes

Are sufficient details of methods and analysis provided to allow replication by others?

Yes

Reviewer Expertise:

heavy metals and their health effects

I confirm that I have read this submission and believe that I have an appropriate level of expertise to confirm that it is of an acceptable scientific standard.

# Reviewer report: Reviewer response for version 2

**DOI:** 10.5256/f1000research.173548.r348711

**Published:** 2024-12-17

**Authors:** Tommaso Filippini

**Affiliations:** 1 University of Modena and Reggio Emilia, Modena, Italy

The manuscript titled "Genotoxic effects in island populations of Cartagena de India's, Colombia due to environmental exposure to mercury and cadmium" presents an interesting study assessing the effects of genotoxicity of cadmium and mercury exposure in a population characterized by higher exposure due to dietary habits (fishing leading to high fish consumption) as well as environmental contamination. The study implemented micronuclei assay for the assessment of genotoxic effects. In general, strengths of the study include clear criteria for identification of study population, the use of established method for assessment of genotoxic effects and correlation analysis and exclusion of some relevant confounding factors (e.g. smoking) . Limitations of the study include the small sample size and the possible exposure to other metals. Authors may expand the reasons for focusing on Cd and Kg among the possible heavy metals with genotoxic effects and characterizing environmental pollution in other areas (e.g. arsenic, lead, etc.).

 Specific comments on the manuscript to clarify the reporting are below:

 - In the Methods, the study period has not been stated. Please add and clarify that the study is cross-sectional.

 - Scale in figure 2 should be added

 - point instead of comma for decimals should be used. Please check text and tables.

 - Table 2 can be omitted, margin data with Table 1, decreasing the number of tables

 - In Table 4, please report 95% confidence intervals along with p values only.

Is the work clearly and accurately presented and does it cite the current literature?

Yes

If applicable, is the statistical analysis and its interpretation appropriate?

Yes

Are all the source data underlying the results available to ensure full reproducibility?

Partly

Is the study design appropriate and is the work technically sound?

Yes

Are the conclusions drawn adequately supported by the results?

Yes

Are sufficient details of methods and analysis provided to allow replication by others?

Yes

Reviewer Expertise:

heavy metals and their health effects

I confirm that I have read this submission and believe that I have an appropriate level of expertise to confirm that it is of an acceptable scientific standard, however I have significant reservations, as outlined above.

# Response

**Published:** 2025-01-08

**Authors:** Eder Cano Pérez

**Affiliations:** University of Cartagena, Colombia

We would like to express our gratitude to the reviewer for the time dedicated to reviewing our study. We highly value all of their comments. Below, we provide our responses to their observations.

 - In the Methods, the study period has not been stated. Please add and clarify that the study is cross-sectional.

 * We agree with the observation made. The type and period of the study have been added in section 2.1 of Materials and Methods

 - Scale in figure 2 should be added

 * We have added scales to the spatial units of interest (study sites: Ararca, Barú, Caño del Oro, and Tierra Bomba).

 - Point instead of comma for decimals should be used. Please check text and tables.

 * We have replaced commas with points for decimals in the tables and text

 - Table 2 can be omitted, margin data with Table 1, decreasing the number of tables

 * We appreciate the reviewer's suggestion. However, since we use a format with independent sections to present the study results (e.g., 3.1.Analysis of metals, 3.2. BMCyt Parameters), we believe it is more practical to maintain Table 1 and Table 2 in their current form rather than merging the information from both tables.

 - In Table 4, please report 95% confidence intervals along with p values only.

 * We appreciate the reviewer's suggestion. We have modified the data reporting as recommended. This modification led us to identify some findings that were initially overlooked by the authors. Two significant negative correlations between blood Cd levels and two DNA damage parameters were discovered. These results have been reported in section 3.2, paragraph 2, and discussed in section 4.0, paragraph 8. To aid in the discussion of these new findings, an additional brief analysis was conducted, which is reported in section 3.1, paragraph 2, in the last three lines.

# Reviewer report: Reviewer response for version 2

**DOI:** 10.5256/f1000research.173548.r333447

**Published:** 2024-12-17

**Authors:** Marina Piscopo

**Affiliations:** 1 University of Naples Federico II, Naples, Italy

The author addressed all my questions. I accept the manuscript in the present form

Is the work clearly and accurately presented and does it cite the current literature?

Partly

If applicable, is the statistical analysis and its interpretation appropriate?

Yes

Are all the source data underlying the results available to ensure full reproducibility?

Yes

Is the study design appropriate and is the work technically sound?

Partly

Are the conclusions drawn adequately supported by the results?

Partly

Are sufficient details of methods and analysis provided to allow replication by others?

Yes

Reviewer Expertise:

Study of the molecular effect of heavy metals and other pollutants on the reproductive health of organism and on red cells

I confirm that I have read this submission and believe that I have an appropriate level of expertise to confirm that it is of an acceptable scientific standard.

# Reviewer report: Reviewer response for version 1

**DOI:** 10.5256/f1000research.169666.r317053

**Published:** 2024-09-14

**Authors:** Marina Piscopo

**Affiliations:** 1 University of Naples Federico II, Naples, Italy

In the article titled “Genotoxic effects in island populations of

 Cartagena de Indias, Colombia due to environmental exposure to mercury

 and cadmium” the authors aimed to evaluate the genotoxic effects of

 environmental exposure to Hg and Cd in populations from the island zone

 of the Cartagena district, Bolívar. They found that cadmiun levels in

 the blood of the study group were within the normal range and comparable

 to those of the control group while significantly higher T-Hg levels

 were registered compared to the control group, surpassing the accepted

 limit. In addition, in this study a higher incidence

 of DNA damage and cell death biomarkers were revealed. Finally,

 significant correlations were observed between total blood Hg levels and

 the frequencies of micronuclei, karyorrhexis, and karyolysis.

 - I find it an interesting and very relevant work since environmental

 pollution is a major concern worldwide. I suggest a major revision,

 especially since the state of the art is not up-to-date and also to try

 to correlate the results obtained with each other for a better

 understanding of the paper.

 The suggestions I would make are as follows:

 Major point

 • There is a different degree of accumulation in blood and semen. I

 suggest to read and quote the following paper in order to amply the

 introduction or discussion[1]

 • Mercury have many effects on the reproductive health of human and

 marina organisms. I suggest to read and quote the following papers in

 order to amply the introduction or discussion[2],[3],[4]

 • Have the authors checked whether the effects of cadmium are seasonal?

 Because e.g. in mussels it is so. [5]

 • Mercury have many effects on hemoglobin and red cells. In addition,

 olive oil and other antioxidant prevent the effect of mercury in red

 cells. I suggest to read and quote the following papers in order to

 amply the introduction or discussion:[6],[7],[8]

 • The results must be commented on and an explanation must also be given

 for the effect of these pollutants on the alterations observed. A

 molecular mechanism must also be better hypothesized

 • try to correlate the results obtained with each other

 Minor point

 • ml must be changed in mL in all manuscript

 • µl must be changed in µL

 • I believe that to make the article more appealing you should include a

 nice color image that summarizes the results, the molecular mechanism

 and the message the authors want to give

 • Better define the limitations of this study

 • English revision required

Is the work clearly and accurately presented and does it cite the current literature?

Partly

If applicable, is the statistical analysis and its interpretation appropriate?

Yes

Are all the source data underlying the results available to ensure full reproducibility?

Yes

Is the study design appropriate and is the work technically sound?

Partly

Are the conclusions drawn adequately supported by the results?

Partly

Are sufficient details of methods and analysis provided to allow replication by others?

Yes

Reviewer Expertise:

Study of the molecular effect of heavy metals and other pollutants on the reproductive health of organism and on red cells

I confirm that I have read this submission and believe that I have an appropriate level of expertise to confirm that it is of an acceptable scientific standard, however I have significant reservations, as outlined above.

## References

[1] : Comparison between Macro and Trace Element Concentrations in Human Semen and Blood Serum in Highly Polluted Areas in Italy. *Int J Environ Res Public Health* .2022;19(18) : 10.3390/ijerph191811635 10.3390/ijerph191811635 36141930 PMC9517217

[2] : Morphological, Gene, and Hormonal Changes in Gonads and In-Creased Micrococcal Nuclease Accessibility of Sperm Chromatin Induced by Mercury. *Biomolecules* .2022;12(1) : 10.3390/biom12010087 10.3390/biom12010087 35053235 PMC8773939

[3] : Spermatozoa Transcriptional Response and Alterations in PL Proteins Properties after Exposure of Mytilus galloprovincialis to Mercury. *Int J Mol Sci* .2021;22(4) : 10.3390/ijms22041618 10.3390/ijms22041618 33562685 PMC7915165

[4] : New Insights into Alterations in PL Proteins Affecting Their Binding to DNA after Exposure of Mytilus galloprovincialis to Mercury-A Possible Risk to Sperm Chromatin Structure?. *Int J Mol Sci* .2021;22(11) : 10.3390/ijms22115893 10.3390/ijms22115893 34072703 PMC8198333

[5] : Seasonal dependence of cadmium molecular effects on Mytilus galloprovincialis (Lamarck, 1819) protamine-like protein properties. *Mol Reprod Dev* .2019;86(10) : 10.1002/mrd.23240 1418-1429 10.1002/mrd.23240 31317589

[6] : Novel Insights into Mercury Effects on Hemoglobin and Membrane Proteins in Human Erythrocytes. *Molecules* .2020;25(14) : 10.3390/molecules25143278 10.3390/molecules25143278 32707650 PMC7397049

[7] : Olive Oil Phenols Prevent Mercury-Induced Phosphatidylserine Exposure and Morphological Changes in Human Erythrocytes Regardless of Their Different Scavenging Activity. *Int J Mol Sci* .2022;23(10) : 10.3390/ijms23105693 10.3390/ijms23105693 35628502 PMC9147954

[8] : Phenol-Rich Feijoa sellowiana (Pineapple Guava) Extracts Protect Human Red Blood Cells from Mercury-Induced Cellular Toxicity. *Antioxidants (Basel)* .2019;8(7) : 10.3390/antiox8070220 10.3390/antiox8070220 31336755 PMC6680458

# Response

**Published:** 2024-10-16

**Authors:** Eder Cano Pérez

**Affiliations:** University of Cartagena, Colombia

General commentary:  We appreciate the reviewer for each of the observations and recommendations provided. Overall, most of these suggestions have been addressed and taken into account to enhance the quality of our article. We hope that the modifications made meet the expectations of both the reviewer and the editorial team, facilitating a successful acceptance process for our manuscript.

 • **There is a different degree of accumulation in blood and semen. I suggest to read and quote the following paper in order to amply the introduction or discussion[1]**

 R/ We appreciate the reviewer’s recommendations. We have opted to include the suggested reference to enhance the introduction of our manuscript

 • **Mercury have many effects on the reproductive health of human and marina organisms. I suggest to read and quote the following papers in order to amply the introduction or discussion[2],[3],[4]**

 R/ We appreciate the reviewer’s recommendations. We have chosen to include some of the suggested references to enhance the introduction of our manuscript.

 • **Have the authors checked whether the effects of cadmium are seasonal? Because e.g. in mussels it is so. [5]**

 R/ We appreciate the reviewer’s observation. In our study, we aimed to assess the genotoxic effects of Hg and Cd in human populations exposed to environmental contamination. However, since our study was a cross-sectional observational cohort, we are unable to determine whether the effects of Cd on these populations vary seasonally.

 • **Mercury have many effects on hemoglobin and red cells. In addition, olive oil and other antioxidant prevent the effect of mercury in red cells. I suggest to read and quote the following papers in order to amply the introduction or discussion:[6],[7],[8]**

 R/ We appreciate the reviewer’s recommendations. We have chosen to include some of the suggested references to enhance the introduction of our manuscript.

 • **The results must be commented on and an explanation must also be given for the effect of these pollutants on the alterations observed. A molecular mechanism must also be better hypothesized**

 R/ We appreciate the reviewer’s recommendation. We have added a paragraph in the discussion of the manuscript outlining the mechanisms by which heavy metals such as Hg can cause DNA damage and lead to the alterations observed in our study (primarily micronuclei). For this, we have consulted relevant scientific literature.

 Minor point

 • **“ml” must be changed in “mL” in all manuscript**

 R/ We appreciate the reviewer’s detailed observation. We have made the correction from "ml" to "mL" throughout the entire document.

 • **µl must be changed in µL**

 R/ We appreciate the reviewer’s detailed observation. We have made the correction from " µl" to " µL" throughout the entire document

 • **Better define the limitations of this study**

 R/ We have revised several aspects of the limitations section to enhance clarity and better articulate our ideas. These improvements aim to provide a more precise understanding of the constraints of our study.

 • **English revision required**

 R/ This study was reviewed by English-speaking experts in the field of toxicology prior to its submission to the journal.
